# Recurrent cholangitis and bacteraemia due to *Edwardsiella tarda*: a case report

**DOI:** 10.1093/omcr/omad148

**Published:** 2024-01-27

**Authors:** Makoto Hasegawa, Yohei Sanmoto

**Affiliations:** Department of Surgery, Takeda General Hospital, Fukushima, Japan; Department of Surgery, Takeda General Hospital, Fukushima, Japan

**Keywords:** Edwardsiella tarda, cholangitis, bacteraemia, carriage, colonization, bacteraemia

## Abstract

*Edwardsiella tarda* is typically isolated from aquatic environments. It rarely causes infections in humans. *Edwardsiella tarda* infections in humans result from the consumption of infected or contaminated food. Here, we present a case of recurrent cholangitis and bacteraemia associated with *E. tarda*. An 82-year-old man with no history of seafood inoculation was admitted to our hospital because of difficulty in moving his body. The patient was diagnosed with cholangitis, and the blood culture revealed the presence of *E. tarda*. The patient underwent bile duct stenting and received antibiotic therapy for 14 days. Forty-four days after discharge, cholangitis recurred, and blood culture again showed the presence of *E. tarda*. The patient underwent bile duct stenting and antibiotic therapy for 11 days. No cholangitis or bacteraemia associated with *E. tarda* was observed in the following 3 years. Our case strongly suggests that colonization with *E. tarda* results in recurrent cholangitis and bacteraemia.

## INTRODUCTION

*Edwardsiella tarda*, a gram-negative facultative anaerobe that is a member of the family Enterobacteriaceae, is typically isolated from aquatic environments and water-dwelling animals [1, 2]. *Edwardsiella tarda* rarely causes infections in humans [1, 2]. However, the prognosis of bacteraemia caused by *E. tarda* is extremely poor, with a mortality rate of 44.6% [1]. It is unclear whether this bacterium persists in the human body. Moreover, reinfection with *E. tarda* has not been reported. Here, we present a case of short-term recurrent cholangitis and bacteraemia associated with *E. tarda*.

## CASE REPORT

An 82-year-old male was brought to the emergency department because of difficulty in moving his body. He had a history of cholangitis, and a bile duct stent was placed a year prior; however, the stent was not replaced because the patient refused to attend the hospital. He has not received any antibiotic treatment for a year since then. He has a history of atrial fibrillation (on rivaroxan medication), chronic heart failure, hypertension, chronic kidney disease, and hyperuricaemia. The patient had no history of seafood inoculation or contact with aquatic environments.

**Figure 1:**
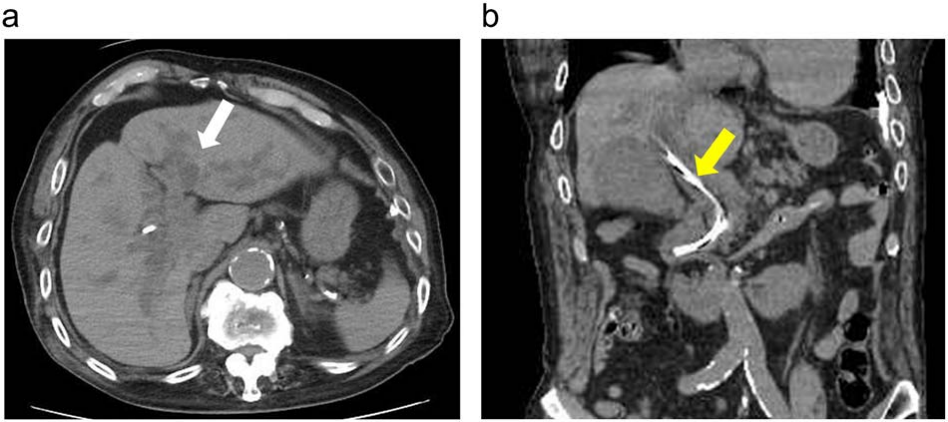
(**a**) Computed tomography showing dilated intrahepatic bile ducts (white arrow). (**b**) Computed tomography showing a bile duct stent (yellow arrow).

On arrival at the emergency department, his temperature was 38.7°C; blood pressure, 116/86 mmHg; pulse rate, 75 beats/min; and oxygen saturation, 97% on room air. The patient was alert and had icteric sclerae but no rash or lymphadenopathy. His abdomen was soft; however, tenderness was noted over the right hypochondrium. Laboratory data revealed an increased inflammatory response and elevated hepatobiliary enzyme levels (Table 1). Computed tomography revealed dilated intrahepatic bile ducts (Fig. 1a and b).

**Table 1:** Laboratory data

Investigations	Blood biochemistry test at 1st cholangitis		Blood biochemistry test at 2nd cholangitis		Reference	
White blood cell	20.4	10^9^/μl	14.9	10^9^/μl	3.3–8.6	10^9^/μl
Neutrophils	92.8	%	-	%	38.5–80.5	%
Haemoglobin	13.2	g/dl	12.3	g/dl	13.7–16.8	g/dl
Platelet count	21.8	10^9^/μl	31.6	10^9^/μl	15.8–34.8	10^9^/μl
Creatinine	1.72	mg/dl	1.60	mg/dl	0.65–1.07	mg/dl
Total bilirubin	8.1	mg/dl	6.3	mg/dl	0.4–1.5	mg/dl
Direct bilirubin	6.7	mg/dl	-	mg/dl	0.0–0.2	mg/dl
Aspartate aminotransferase	77	U/l	77	U/l	13–30	U/l
Alanine aminotransferase	95	U/l	77	U/l	10–42	U/l
Alkaline phosphatase	672	U/l	858	U/l	38–113	U/l
Gamma glutamyl transferase	299	U/l	313	U/l	13–64	U/l
Lactate hydrogenase	259	U/l	235	U/l	124–222	U/l
Amylase	68	U/l	135	U/l	44–132	U/l
C-reactive protein	16.95	mg/dl	10.53	mg/dl	0.00–0.14	mg/dl
International normalized ratio	1.24		1.13		0.88–1.12	
Activatedpartialthromboplastintime	38.2	s	33.5	s	23.0–39.0	s

**Figure 2:**
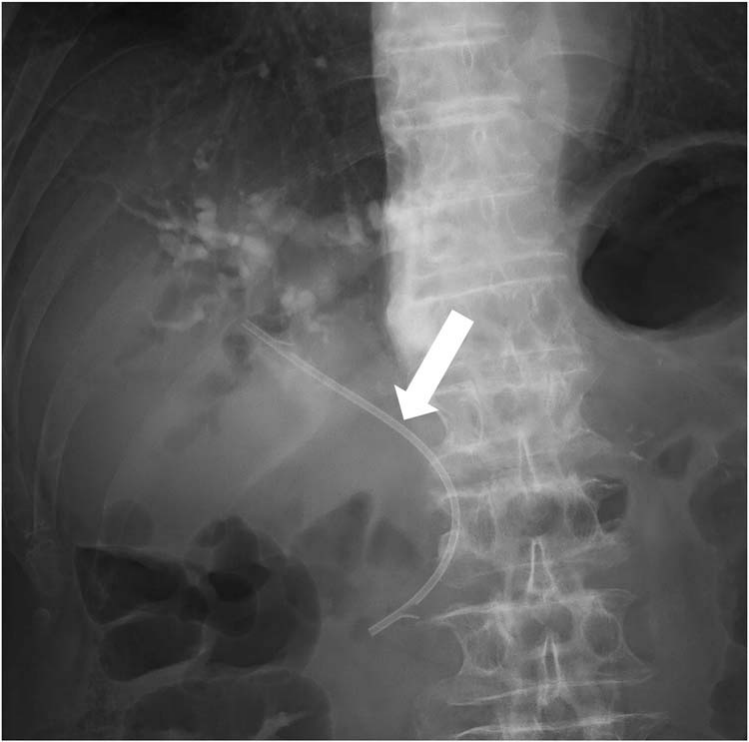
In cholangiography, biliary stent was replaced again (Flexima™ Biliary Stent System 7Fr 10 cm; Boston Scientific) (white arrow).

We diagnosed cholangitis associated with stent obstruction and performed endoscopic retrograde cholangiopancreatography to replace the bile duct stent (Flexima™ Biliary Stent System 7Fr 10 cm; Boston Scientific, Marlborough, Massachusetts, United States) (Fig. 2). Empiric antibiotic therapy with meropenem (1 g IV q12h) was initiated after two sets of blood cultures were obtained and *Edwardsiella tarda* and *Aeromonas caviae* were detected. The patient was admitted to the high care unit because of progressive decrease in blood pressure and the need for vasopressors. Afterwards, the infection was under control, so on day 8 the antibiotic was changed to Augmentin (amoxicillin/clavulanate; 500/125 mg PO q8h) which has good bioavailability and continued for 7 days (the total duration of antibiotic treatment was 14 days). The patient was discharged on day 12.

Forty-four days after discharge, the patient was admitted to the emergency department, with symptoms similar to those observed previously. On arrival at the emergency department, his temperature was 39.4°C; blood pressure, 83/54 mmHg; pulse rate, 71 beats/min; and oxygen saturation, 90% on room air. The patient had a Glasgow Coma Scale score of E4V3M5, with impaired consciousness and icteric sclerae but no rash or lymphadenopathy. The abdomen was soft; however, the right hypochondrium was tender. Laboratory data revealed an increased inflammatory response and elevated hepatobiliary enzyme levels (Table 1). Computed tomography revealed dilated intrahepatic bile ducts. We diagnosed cholangitis associated with stent obstruction and performed endoscopic retrograde cholangiopancreatography; the biliary stent was replaced again (Flexima™ Biliary Stent System 7Fr 10 cm; Boston Scientific). Empiric antibiotic therapy with tazobactam/piperacillin (4.5 g IV q8h) was initiated after two sets of blood cultures were obtained, and *E. tarda* was detected. The sensitivity results showed that ampicillin and cefaclor changed from sensitive (in the previous culture results) to intermediate and resistant (Table 2). The amylase level increased from 135 U/l on admission to 1036 U/l on day 3, suggesting possible gallstone pancreatitis. Endoscopic sphincterotomy was performed, and bile duct stones were excised. On day 5, a suspected drug rash caused by tazobactam/piperacillin occurred, and the antibiotic was changed to levofloxacin (500 mg × 1 IV, followed by 250 mg q24h), which was continued for 7 days (the total duration of antibiotic treatment was 11 days). The patient was discharged on day 11.

**Table 2:** Antibiotic susceptibility of *Edwardsiella tarda* from blood culture

Antibiotic	MIC of 1st detected *E. tarda* (μg/ml)	Sensitivity	MIC of 2nd detected *E. tarda* (μg/ml)	Sensitivity
Piperacillin	≦8	sensitive	≦8	sensitive
Ampicillin	≦8	sensitive	16	intermediate
Sulbactam/Ampicillin	≦8	sensitive	≦8	sensitive
Cefazolin	≦4	sensitive	8	sensitive
Cefaclor	≦8	sensitive	>16	resistance
Cefotiam	≦8	sensitive	≦8	sensitive
Ceftazidime	≦4	sensitive	≦4	sensitive
Cefmetazole	≦8	sensitive	≦8	sensitive
Sulbactam/Cefoperazone	≦16	sensitive	≦16	sensitive
Imipenem/Cilastatin	≦1	sensitive	≦1	sensitive
Flomoxef	≦8	sensitive	≦8	sensitive
Minocycline	≦2	sensitive	≦2	sensitive
Gegntamicin	≦2	sensitive	≦2	sensitive
Amikacin	≦4	sensitive	≦4	sensitive
Sulfamethoxazole Trimethoprim	≦2	sensitive	≦2	sensitive
Levofloxacin	≦0.5	sensitive	≦0.5	sensitive
Ceftriaxone	≦1	sensitive	≦1	sensitive
Cefepime	≦2	sensitive	≦2	sensitive
Meropenem	≦1	sensitive	≦1	sensitive
Piperacillin/Tazobactam	≦16	sensitive	≦16	sensitive

MIC, minimum inhibitory concentration.

Since then, the patient has not experienced a recurrence of cholangitis or bacteraemia due to *E. tarda* for 3 years.

## DISCUSSION

*Edwardsiella tarda* is a relatively rare human pathogen commonly found in freshwater or brackish water environments, such as estuaries [1, 2]. *Edwardsiella tarda* is detected in 0.02% of blood cultures [3] and rarely causes infections in humans. *Edwardsiella tarda* infections in humans result from the consumption of infected or contaminated food, such as fish [1]. The gastrointestinal tract is the most commonly affected organ by *E. tarda* [2, 4]. Extraintestinal infections, such as cholangitis, have been reported less frequently [1].

As discussed below, this patient was considered to be at high risk of cholangitis and bacteraemia associated with *E. tarda*. Age ≥65 years is significantly associated with an increased risk of *E. tarda* bacteraemia [3], and the main underlying conditions in *E. tarda* bacteraemia are hepatobiliary disease (liver cirrhosis, gallbladder stones, and ethanol abuse), malignancy, and iron overload status (sickle cell disease, leukaemia, and neonatal condition) [1, 4].

In this case, the patient had recurrent cholangitis and bacteraemia associated with *E. tarda* within a short period, strongly suggesting colonization with *E. tarda*. To the best of our knowledge, this is the first reported case of recurrent *E. tarda* bacteraemia and cholangitis. Little is known about the current prevalence of *E. tarda* colonization. A study conducted in the 1970s [5] reported that only 26 out of 353 600 (0.007%) Japanese individuals were healthy carriers of *E. tarda* in their digestive tract. Based on the duration of antibiotic treatment for severe cholangitis, treatment for 4–7 days with clinical resolution has been suggested [6]. The median duration of antibiotic treatment for *E. tarda*-associated bacteraemia is reported to be 12 days [3], and the duration of our treatment for *E. tarda*-associated bacteraemia was considered sufficient (14 days).

## CONCLUSION

Here, we report a case of recurrent cholangitis and bacteraemia caused by *E. tarda*. The colonization rate of *E. tarda* in the human body is low; however, our results strongly suggest that *E. tarda* colonization causes recurrent cholangitis and bacteraemia.

## CONFLICT OF INTEREST STATEMENT

The authors declare that they have no competing.

## FUNDING

This research did not receive any specific grants from funding agencies in the public, commercial, or not-for-profit sectors.

## ETHICAL APPROVAL

This case report did not require review by the relevant Ethics Committees.

## CONSENT

The patient provided informed consent for publication of the report and associated images.

## GUARANTOR

Makoto Hasegawa.
